# Hematological, Biochemical, and Serological Findings in Healthy Canine Blood Donors after the Administration of CaniLeish® Vaccine

**DOI:** 10.1155/2016/4601893

**Published:** 2016-05-22

**Authors:** Chiara Starita, Alessandra Gavazza, George Lubas

**Affiliations:** Department of Veterinary Sciences, University of Pisa, Via Livornese Lato Monte, San Piero a Grado, 56122 Pisa, Italy

## Abstract

The aim of the study was to evaluate hematological, biochemical, and serological findings in healthy canine blood donors after the administration of CaniLeish® vaccine. Twenty-seven client-owned dogs were included in the study and arranged into 3 groups according to the vaccination stage. Complete blood count (CBC) with blood smear examination, serum biochemical profile (SBP), serum protein electrophoresis (SPE), and serological tests for* L. infantum* were performed at different times. Additionally, in a subgroup of dogs IgA, IgM, and IgG were quantified. No statistical significance for CBC and SBP was found. In 10.7% of cases slight hyperproteinemia occurred. In SPE absolute values *β*-1-globulins (Group 2 and Group 2-3) and *β*-2-globulins (Group 3) were found modified (*P* < 0.05). IgG values were statistically different (*P* < 0.05) 6–8 months after the third immunisation (Group 2) and IgM and IgG values were statistically different after 2 months (Group 3). IFAT positive samples were 20.8% (Group 1), 15.0% (Group 2), and 52.8% (Group 3). Speed Leish K*™* tests were always negative. The modifications found were probably attributed to the development of immune or inflammatory response due to the vaccine. Administration of CaniLeish vaccine in canine blood donors could be a safe practice and did not affect their health status.

## 1. Introduction 

Canine leishmaniosis is caused by an intracellular protozoan called* Leishmania infantum* transmitted by sand flies of the genus* Phlebotomus.* The progression from infection to clinical disease occurs if the canine cell-mediated immune response is inadequate and the parasite increases in number within macrophages in many organs and tissues [1].

The prevention of canine leishmaniosis requires a combined approach including measures focused both on dogs and on environment [2–4]. A canine vaccine that modulates cell-mediated immune response against the protozoan has been available in Italy since 2012 (CaniLeish, Virbac, France). It reduces the risk of developing, after the contact with the parasite, from an active infection to a symptomatic disease [5–7]. In addition, it may help those dogs that get infected despite vaccination, as suggested by a recent study using xenodiagnosis, since disease severity appears to be generally associated with high parasite loads in the skin and their infectivity [8].

Hence, the control of leishmaniosis is particularly important in canine blood donors because the risk of transmission of infectious agents through transfused blood products from blood donors, that are carriers of infection, is demonstrated [9–11]. Overall, protozoan diseases have long incubation periods, subclinical persistence in infected animals, and likelihood of remaining viable in bloodstocks [10, 11].

The recently revised Italian guidelines about veterinary transfusion medicine established by the Ministry of Health [12] stated that blood donor dogs should be healthy animals and should undergo complete clinical examination and laboratory tests including hematobiochemical profile and serological assay, using IFAT, or PCR for* Leishmania infantum*,* Ehrlichia canis*,* Anaplasma phagocytophilum*,* Rickettsia rickettsii,* and* Babesia canis* [13].

In order to increase the prevention of leishmaniosis in blood donors, the vaccine against leishmaniosis could be used. To the author's knowledge no data has been published about the evaluation of hematological and biochemical findings after administration of CaniLeish. Therefore, the aim of this study was to evaluate hematological, biochemical, and serological findings in a group of healthy dogs participating in a voluntary blood donor program at the Transfusion Veterinary Centre, University of Pisa, receiving a full coverage of immunisation with CaniLeish.

## 2. Materials and Methods

### 2.1. Selection Criteria

The study took place between February 2013 and July 2014 in the area of northern Tuscany, Italy. Twenty-seven client-owned dogs participating in a voluntary blood donor program at the University of Pisa were included (written consent was previously collected from all the owners). The following selection criteria were used to include dogs as blood donors: Dog Erythrocyte Antigen (DEA) 1 negative, absence of any clinical signs or symptoms of disease, values of complete blood count (CBC) (ProCyte Dx®, Idexx, Italy) and blood smear examination, serum biochemical profile (SBP) (total protein, albumin, urea, alkaline phosphatase, and alanine aminotransferase) (Liasys®, Assel, Italy), and serum protein electrophoresis (SPE) in agarose gel (Pretty®, Interlab, Italy) within the reference ranges of the Veterinary Clinical Pathology University Laboratory: negative serology for* Leishmania infantum* using immunofluorescence antibody test (IFAT) [14] and Speed Leish K*™* test, negative serology for* Ehrlichia canis* and* Anaplasma phagocytophilum* using IFAT [15]. Any serological positivity titre starting from 1 : 40 was an exclusion cause from the blood donors program. Moreover, all dogs received a regular protection against ectoparasites, both repellent and/or antifeeding drugs applied locally.

### 2.2. Vaccine Administration

Lyophilized CaniLeish vaccine (Virbac, France) stored at +4/+10°C was reconstituted with its solvent (approximately 1 mL) and administered subcutaneously in the withers region followed by a gentle massage of the site. Dogs were monitored for 30 minutes in order to observe the onset of possible anaphylactic reactions. The owners were advised to report to the authors any suspected reaction or adverse effect that might occur and in that case they would have undergone a control. The vaccine was administered according to the protocol indicated in the manufacturer's instructions: first cycle of 3 inoculations, each of them every 3 weeks, and annual boosters for further administration.

### 2.3. Study Design

Twenty-seven canine blood donors (17 females, 10 males; 14 Boxers, 8 mixed breeds, 3 Golden Retrievers, 1 Weimaraner, 1 Border Collie; 2–7 years old) were included in the study. Dogs were divided into three groups according to the vaccination stage. Group 1 included 6 dogs that underwent first and second annual boosters because they had already completed the first cycle of immunisation by their referring veterinarian. Group 2 included 12 dogs, which underwent the first cycle of immunisation and first annual booster. Group 3 included 9 dogs, which underwent only the first cycle of immunisation. The times (*T*) in days of controls were *T*0 (first immunisation), *T*21 (second immunisation), *T*42 (third immunisation), *T*100 (two months after the third immunisation), *T*250 (6–8 months after the third immunisation), *T*405 (first annual booster), and *T*770 (second annual booster). About 10 mL of blood was withdrawn from the jugular or cephalic vein for laboratory analysis. For Group 1 only serological assays (IFAT* L. infantum* and Speed Leish K) were performed at *T*0, *T*250, *T*405, and *T*770. For Group 2, CBC with blood smear examination, serum biochemical profile (total protein, albumin, urea, alkaline phosphatase, and alanine aminotransferase), serum electrophoresis, IFAT for* L. infantum,* and Speed Leish K were provided at *T*0, *T*21, *T*42, *T*250, and *T*405. In Group 3, the same laboratory tests of Group 2 were performed at *T*0, *T*21, *T*42, and *T*100. Group 2 and Group 3 were evaluated together as Group 2-3 whenever possible, for comparison purposes. Moreover, for a subgroup of dogs (10/12 of Group 2 and 7/9 of Group 3) IgA (immunoglobulin), IgM, and IgG fractions were quantified, respectively, at *T*0–*T*100 and *T*0–*T*250 using the method described by Tvarijonaviciute [16]. Briefly, commercial kits (Olympus Europe GmbH) were run on an automatic analyzer (Olympus AU600, Olympus Europe GmbH, Hamburg, Germany) following the manufacturer's instruction.

### 2.4. Statistics

Data distribution was assessed through the D'Agostino-Pearson test. The Kruskal-Wallis test was performed for CBC, SBP, and SPE data, while for immunoglobulin the Wilcoxon test was provided. For all tests, significance was set as *P* < 0.05. Statistical analysis was performed using commercial software (MedCalc® Software v.14.8.1.0, Mariakerke, Belgium).

## 3. Results 

### 3.1. Hematobiochemical Analysis

No statistical significance for data from CBC and SBP was found (data not shown). However, slight hyperproteinemia up to 8.4 g/dL (reference range 5.8–7.8 g/dL) occurred in 10.7% of cases. Results from SPE are shown in Table 1 as absolute values using median and 95% confidence interval. Our data show a statistical significance (*P* < 0.05) of *β*-1-globulins in Group 2 and Group 2-3 and of *β*-2-globulins in Group 3. Tables 2 and 3 report the results of the quantification of IgA, IgM, and IgG in Groups 2 and 3, respectively. The IgG values were statistically different (*P* < 0.05) at *T*250 for Group 2, while IgM and IgG values were statistically different (*P* < 0.05) at *T*100 in Group 3.

### 3.2. Serological Tests

**Figure 1:**
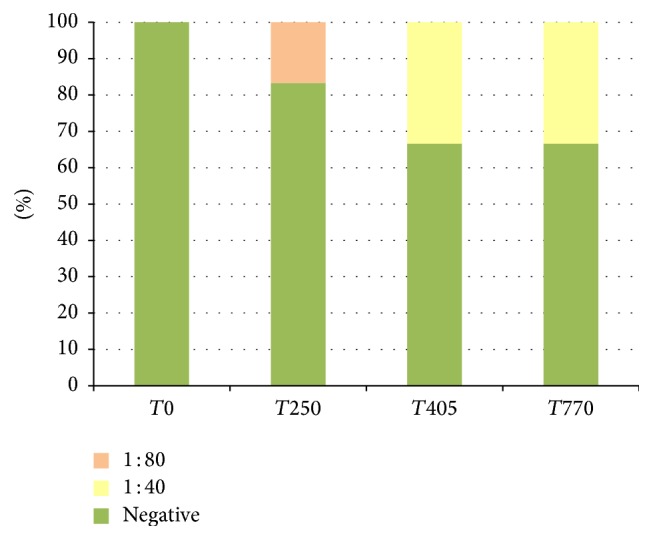
IFAT positivity (%), Group 1.

**Figure 2:**
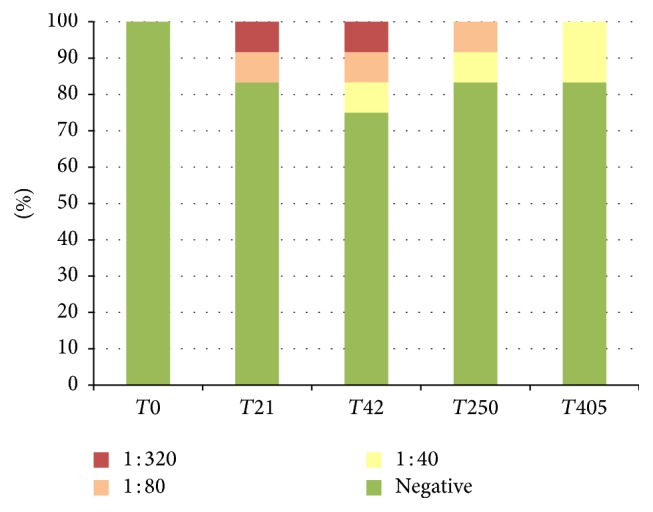
IFAT positivity (%), Group 2.

**Figure 3:**
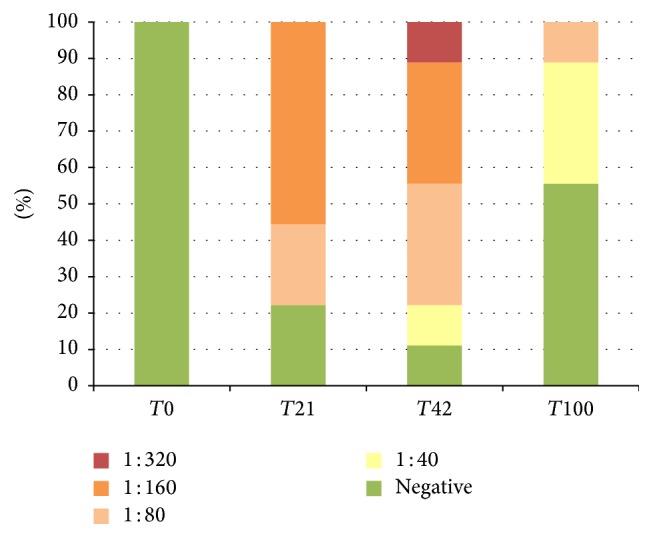
IFAT positivity (%), Group 3.

The results of IFAT are reported in Table 4 (and Figures 1, 2, and 3). In Group 1, 20.8% of canine samples were positive at low titres (up to 1 : 80), in Group 2, 15.0% of samples were positive at rather low titres (mostly up to 1 : 80, one dog up to 1 : 320), and, in Group 3, 52.8% of samples were positive at rather low and high titres (mostly 1 : 80 and 1 : 160, one dog up to 1 : 320). In detail, in Group 2 two dogs at *T*21 and three dogs at *T*100 (during the initial immunisation) showed variable titres up to 1 : 320. At the following monitoring, only one dog was continuously positive for all the observations. In Group 3 most of the dogs were showing positive titre for IFAT after the initial immunisation (*T*21) and this trend was observed for the other two collection points as well. All Speed Leish K tests were negative throughout the study period.

## 4. Discussion

In the present study the serological status of dogs after the administration of CaniLeish was monitored up to the second annual booster. No publications have so-far assessed long-term IFAT titres modifications in vaccinated dogs. The positivity was generally low and the trend is various for all dogs. The prevalence recorded was 20.8% in Group 1, 15.0% in Group 2, and 52.8% in Group 3. Many previous studies reported an increase of IFAT titres after vaccination. Sagols et al. demonstrated the seroconversion of dogs vaccinated from 2 weeks to 4 months after the third shot and the gradual decrease of titres in the following monitoring [17]. Moreno et al. indicated the increase of IFAT titres in all 20 vaccinated dogs at weeks 8 and 12 (maximum titre 1 : 500) after the first injection and one dog still had a titre of 1 : 200 at week 30 [6]. All dogs became negative at IFAT (titre <1 : 200) at week 42. Furthermore, in another study Sagols et al. tested 31 vaccinated dogs after 3-4 weeks from the first annual booster evidencing the increase of IFAT titres [18]. Recently, Sagols et al. investigated the follow-up of the humoral immune response after the first annual booster recording an evolution of IFAT titres similar to the transient profile obtained after the primary vaccination [19]. However, IFAT results should be interpreted with caution in vaccinated dogs, because they are obtained from a nonspecific test that detects all IgG antibodies against the whole parasite, both due to the contact with the parasite and due to the development of immunological response. According to previous studies by Sagols et al. all Speed Leish K assays remained negative, so the antibodies detected by IFAT technique were not identified by this test (immunochromatographic anti-kinesin antibody test) [17–19]. Therefore, Sagols et al. demonstrated that this rapid test is reliable for detecting antibodies due to the contact with the parasite. On the other hand, Solano-Gallego et al. have indicated that the sensitivity of rapid serological tests used for dog screening prior to vaccination with CaniLeish is rather low [20]. Thus, it is likely that some dogs will be already* Leishmania*-infected at the time of vaccination, and this will be a complicating factor for assessing vaccine efficacy in the field. Moreover, the EFSA AHAW panel shows that antibodies elicited on vaccinated dogs cannot be discriminated with current serological methods [21]. Until the advent of vaccination in Europe, standard guidelines suggested that IFAT titres of at least 4 times the laboratory cut-off level were indicative of the disease and that levels between the threshold and 4 times the threshold raised a suspicion of the disease [22]. Nowadays, the literature agrees that the most useful diagnostic approaches for investigation of infection in sick and clinically healthy infected dogs include both the detection of specific serum anti-*Leishmania* antibodies by quantitative serological techniques and the demonstration of the parasite DNA in tissues by applying specific molecular techniques [1, 4, 21, 22].

To the author's knowledge, no previous data about the follow-up and monitoring of dogs vaccinated through hematobiochemical and SPE analysis were published. Our results for SPE showed the increase of few globulins fractions (*β*-globulins) that could be attributed to the immune response induced by the vaccine. Proteins that migrate in each fraction should be investigated individually, but this was not the goal of this study. Additionally, modifications observed in Ig quantification could be attributed to the immune stimulation induced by the vaccine. The significant increase of total IgG anti-*Leishmania* antibodies after vaccination with CaniLeish is also demonstrated in other studies [5, 7].

In our study the limits were the low number of dogs included, their different vaccination stages, the lack of PCR analysis of blood to rule out the possibility of transmission of* L. infantum* through bloodstocks, and the lack of ELISA testing for dosing the level of IgG1 and IgG2 to assess the type of immune response. The study is still continuing and further data will be published.

## 5. Conclusions

In conclusion, the adoption of the vaccine CaniLeish in a group of blood donors, typically healthy dogs as they are frequently monitored all year long, could be a safe practice. After vaccination there are only minimal modifications in total protein contents, some globulins fractions, IgM and IgG, and mild increase of IFAT titres.

## Figures and Tables

**Table 1:** Major serum proteins and electrophoresis fractions reported as absolute values in Groups 2 and 3.

Analyte^§^	Time	Group 2 median (*n* = 12)	95% CI median	Group 3 median (*n* = 9)	95% CI median	Group 2-3 median (*n* = 21)	95% CI median
Total protein (5.8–7.8 g/dL)	*T*0	7.2	6.6–7.8	6.5	6.3–6.8	6.7	6.5–7.2
	*T*21	7.1	6.8–7.6	6.8	6.0–7.7	7.0	6.7–7.5
	*T*42	7.1	6.6–7.6	6.5	6.2–7.4	6.8	6.5–7.3
	*T*100	NT	NT	6.8	6.5–7.5	NT	NT
	*T*250	6.6	6.5–7.8	NT	NT	NT	NT
	*T*405	7.2	6.6–7.5	NT	NT	NT	NT

Albumin (2.6–4.1 g/dL)	*T*0	3.5	3.2–4.1	3.4	3.1–3.5	3.4	3.3–3.5
	*T*21	3.5	3.2–3.7	3.3	2.9–3.9	3.4	3.3–3.7
	*T*42	3.5	3.3–3.7	3.3	3.0–3.7	3.4	3.2–3.6
	*T*100	NT	NT	3.3	3.1–3.7	NT	NT
	*T*250	3.4	3.1–3.6	NT	NT	NT	NT
	*T*405	3.3	3.1–3.5	NT	NT	NT	NT

Globulins (2.5–4.5 g/dL)	*T*0	3.6	3.0–4.2	3.2	2.8–3.6	3.2	3.0–3.6
	*T*21	3.7	3.5–3.9	3.4	3.0–3.8	3.5	3.3–3.8
	*T*42	3.5	3.2–4.0	3.3	3.2–3.7	3.5	3.3–3.7
	*T*100	NT	NT	3.6	3.2–4.0	NT	NT
	*T*250	3.5	3.1–4.4	NT	NT	NT	NT
	*T*405	3.9	3.5–4.3	NT	NT	NT	NT

Alpha-1-globulins (0.1–0.3 g/dL)	*T*0	0.3	0.32–0.3	0.2	0.2–0.3	0.3	0.2–0.3
	*T*21	0.3	0.2–0.3	0.2	0.2–0.23	0.2	0.2–0.3
	*T*42	0.3	0.2–0.3	0.2	0.2–0.3	0.2	0.2–0.3
	*T*100	NT	NT	0.2	0.2–0.3	NT	NT
	*T*250	0.2	0.2–0.3	NT	NT	NT	NT
	*T*405	0.3	0.2–0.3	NT	NT	NT	NT

Alpha-2-globulins (0.6–1.4 g/dL)	*T*0	1.0	0.7–1.2	1.0	0.8–1.1	1.0	0.9–1.1
	*T*21	1.0	0.9–1.0	1.1	1.0–1.3	1.0	0.9–1.1
	*T*42	0.9	0.8–1.0	1.1	1.0–1.1	1.0	0.9–1.1
	*T*100	NT	NT	1.0	0.9–1.3	NT	NT
	*T*250	1.0	0.9–1.2	NT	NT	NT	NT
	*T*405	1.1	1.0–1.2	NT	NT	NT	NT

Beta-1-globulins (0.3–1.0 g/dL)	*T*0	0.6^*∗*^	0.4–0.8	0.6	0.3–0.9	0.6^*∗*^	0.5–0.8
	*T*21	0.5^*∗*^	0.4–0.5	0.5	0.4–0.6	0.5^*∗*^	0.4–0.5
	*T*42	0.5^*∗*^	0.4–0.5	0.4	0.4–0.5	0.5^*∗*^	0.4–0.5
	*T*100	NT	NT	0.4	0.3–0.7	NT	NT
	*T*250	0.9^*∗*^	0.5–1.0	NT	NT	NT	NT
	*T*405	0.9^*∗*^	0.4–1.1	NT	NT	NT	NT

Beta-2-globulins (0.4–1.1 g/dL)	*T*0	1.0	0.3–1.8	0.7^*∗*^	0.7–0.9	0.7	0.7–1.0
	*T*21	1.1	1.0–1.2	1.0^*∗*^	0.7–1.1	1.0	0.9–1.1
	*T*42	1.0	0.9–1.2	1.0^*∗*^	0.9–1.1	1.0	1.0–1.1
	*T*100	NT	NT	1.2^*∗*^	0.8–1.4	NT	NT
	*T*250	0.9	0.6–1.1	NT	NT	NT	NT
	*T*405	0.8	0.7–1.0	NT	NT	NT	NT

Gamma globulins (0.4–0.9 g/dL)	*T*0	0.9	0.6–1.1	0.6	0.4–0.7	0.7	0.5–0.7
	*T*21	0.9	0.8–0.9	0.7	0.5–0.9	0.8	0.7–0.9
	*T*42	0.9	0.6–1.0	0.6	0.5–0.7	0.7	0.6–0.9
	*T*100	NT	NT	0.7	0.5–0.9	NT	NT
	*T*250	0.7	0.5–1.0	NT	NT	NT	NT
	*T*405	0.8	0.6–0.9	NT	NT	NT	NT

^§^Values in brackets are reference ranges.

NT = not tested.

^*∗*^Kruskal-Wallis test was *P* > 0.05 for all time comparison for each group except where the asterisk next to the median values is reported (*P* < 0.05).

**Table 2:** Immunoglobulin concentration in Group 2 (10 dogs).

Analyte^§^	Time	Median	95% CI median
IgA (0.1–1.8 mg/dL)	*T*0	11.3	0.1–18.3
	*T*250	8.8	1.3–18.0

IgM (61–99 mg/dL)	*T*0	184.5	113.8–211.6
	*T*250	180.5	119.0–212.9

IgG (323–659 mg/dL)	*T*0	411.5^*∗*^	317.3–466.7
	*T*250	444.5^*∗*^	352.8–467.5

^§^Values in brackets are reference ranges.

*∗* = Wilcoxon test *P* < 0.05.

**Table 3:** Immunoglobulin concentration in Group 3 (7 dogs).

Analyte^§^	Time	Median	95% CI median
IgA (0.1–1.8 mg/dL)	*T*0	8.1	3.1–14.1
	*T*100	10.8	3.7–14.5

IgM (61–99 mg/dL)	*T*0	110.0^*∗*^	91.5–136.6
	*T*100	140.0^*∗*^	116.9–158.6

IgG (323–659 mg/dL)	*T*0	497.0^*∗*^	448.9–566.5
	*T*100	575.0^*∗*^	504.0–704.1

^§^Values in brackets are reference ranges.

*∗* = Wilcoxon test *P* < 0.05.

**Table 4:** IFAT *L. infantum* for the three groups of dogs.

Time	Group 1 (*n*)	Group 2 (*n*)	Group 3 (*n*)
*T*0	Negative (6)	Negative (12)	Negative (9)

*T*21	Not tested	Negative (10) 1 : 80 (1) 1 : 320 (1)	Negative (2) 1 : 80 (2) 1 : 160 (5)

*T*42	Not tested	Negative (9) 1 : 40 (1) 1 : 80 (1) 1 : 320 (1)	Negative (1) 1 : 40 (1) 1 : 80 (3) 1 : 160 (3) 1 : 320 (1)

*T*100	Not tested	Not tested	Negative (5) 1 : 40 (3) 1 : 80 (1)

*T*250	Negative (5) 1 : 80 (1)	Negative (10) 1 : 40 (1) 1 : 80 (1)	Not tested

*T*405	Negative (4) 1 : 40 (2)	Negative (10) 1 : 40 (2)	Not tested

*T*770	Negative (4) 1 : 40 (2)	Not tested	Not tested
